# Bio-Inspired Sutures: Using Finite Element Analysis to Parameterize the Mechanical Response of Dovetail Sutures in Simulated Bending of a Curved Structure

**DOI:** 10.3390/biomimetics7020082

**Published:** 2022-06-16

**Authors:** Melissa M. Gibbons, Diana A. Chen

**Affiliations:** 1 Department of Mechanical Engineering, University of San Diego, San Diego, CA 92110, USA; 2 Department of Integrated Engineering, University of San Diego, San Diego, CA 92110, USA; dianachen@sandiego.edu

**Keywords:** bio-inspired, suture, mechanical properties, parameterization, finite element model, curved structure, displacement controlled, helmet design

## Abstract

Many animals have protective anatomical structures that allow for growth and flexibility; these structures contain thin seams called sutures that help the structure to absorb impacts. In this study, we parameterized the stiffness and toughness of a curved archway structure based on three geometric properties of a suture through finite element, quasi-static, three-point bending simulations. Each archway consisted of two symmetric pieces linked by a dovetail suture tab design. The three parameters included suture tab radii (1–5 mm), tangent lengths (0–20 mm), and contact angles (0–40°). In the simulations, a steel indenter was displaced 6.5 mm to induce progressive tab disengagement. Sutures with large contact angles and large tangent lengths generally led to stiffer and tougher structures. Sutures with a small tab radius exhibited the most sensitivity to the input parameters, and the smallest tab radius led to the stiffest and toughest archways. Results suggested that it was a combination of the largest number of tab repeats with the largest possible contact surface area that improved the mechanical response of the archway. The study revealed several suture geometries that hold significant promise, which can aid in the development of hemispherical 3D structures for dynamic impact applications.

## 1. Introduction

Biological materials have been optimized by Nature to perform a variety of structural functions, including protection, aggression, and support [1]. The efficiency of these natural designs, following the principle of form follows function [2], have led to entire fields of bio-inspired research seeking to mimic the structure of biological materials and organisms in engineering applications [3]. In doing so, biomimetic engineering studies have analyzed biological systems to determine the building blocks of certain morphologies that drive behavioral responses to various system inputs [4]. In mechanical studies, parameterizing these building blocks as spring–mass–damper systems has allowed engineers to better examine the mechanical response of different forms [5,6]. Our study draws inspiration from Nature to augment energy-absorbing engineered materials, such as those used in helmets, with geometric features found in biological systems, such as sutures that conjoin stiff and tough materials.

Sutures are one example of a natural design feature found in a wide variety of species, including in human skulls [7], the beaks of woodpeckers [8], and in the carapace of turtles [9], in which building blocks of a stiff material such as bone are joined at the seams by a much softer interfacial material. The turtle carapace (the dorsal component of the shell) is comprised of hard building blocks that serve as an extremely strong exoskeleton in case of an attack, while retaining the flexibility needed for growth, locomotion, and respiration [10]. The mineralized ribs that provide the primary stiffness of the structure are connected to one another by unmineralized fibrous sutures that are much more compliant. It has been found that biological sutures increase the overall flexibility and fracture toughness of the structure while maintaining the structural integrity provided by the stiffer building blocks [11]. In mechanics, toughness measures a material’s ability to absorb energy before failing and is given by the strain energy density determined by integrating an experimental stress–strain curve. For example, as a semi-brittle material, bone is not particularly tough [12]; it is, however, very stiff (i.e., it has a large Young’s modulus) [13]. Experimental testing on goat cranial bone has shown that the geometric features of the suture morphology, including the degree of interdigitation and the length and width of the suture, affect the mechanical response of the material (i.e., toughness and stiffness) [14].

Several previous studies have analytically and experimentally examined the effect of trapezoidal suture morphologies on the mechanical response (e.g., load transmission, deformation mechanisms, stiffness, strength, and toughness) to in-plane loading. Analytical studies have examined the geometries under tension normal to the suture axis, under tension parallel to the suture axis, and under simple shear [15]. Experimental studies have examined the geometries under quasi-static tensile loading normal to the suture axis using 3D-printed test pieces [16]. A soft interfacial material was included in these studies, and the ratio of the Young’s modulus of the bulk material to the Young’s modulus of the soft interfacial material varied from 10 to 1000. The composite structure with a suture interface was found to have greater stiffness, strength, and toughness than a composite structure with a flat interface. Additionally, the values of the stiffness, strength, and toughness varied by as much as an order of magnitude with a suture compared to without, providing an opportunity to tune the geometric features to produce a specific mechanical response. 

Other studies have focused on rounded suture morphologies without a soft interfacial material to isolate the effects of the contact mechanics and the suture geometry. Malik et al. (2017) analytically studied round jigsaw-like sutures under tension normal to the suture axis and validated their results by mechanically testing 3D-printed test pieces [17]. In their study, the suture tab size was controlled by the radius of the arc, and the degree of interlocking was controlled by the contact angle, which ranged from 0° to 60°. They found that optimal (i.e., maximum) stiffness and strength were achieved when the contact surface between tabs was frictionless and the interlocking angle of the tabs was about 13°. The chance of tab fracture increased with increasing friction and contact angle, as increases in these values increased the maximum tab stresses; however, of the two, frictional stress dominated and was the limiting design factor. In a subsequent study, the contact area was increased by including a dovetail-like feature in the suture in which a straight-line segment was introduced between the protruding and recessed tabs [18]. While the addition of the dovetail-like feature increased the toughness of the structure substantially, indicating that increasing the contact area was mechanically beneficial, minimal friction and a small contact angle were still necessary to avoid tab fracture.

One potential application of these sutures is in the design of helmets that utilize geometric connections, rather than relying entirely on new engineering materials, to absorb high-impact loads. As such, structural toughness is critical for absorbing energy to prevent the impact loads from being transferred to the skull, while structural stiffness is important for resisting deformation to prevent the helmet itself from injuring the user. However, the optimal suture morphology in curved structures loaded out-of-plane cannot be extrapolated from the previous studies, which were limited to in-plane loading. We draw additional inspiration from Nature from the outer structure of viruses, known as capsids. Two lessons that we can draw from viral capsids for the helmet application are (1) the measured structural responses of a roughly spherical shape to out-of-plane loading, and (2) their ability to adapt their overall size using a modular assembly of building blocks.

The response of the helmet structure based on where it is impacted (surface or suture) is particularly relevant. It can be estimated from prior studies on roughly spherical (icosahedral) viral capsids, in which the mechanical response to out-of-plane loading was measured by indenting different areas of the capsid structure (vertices, edges, or faces) [19,20]. While the sizes of icosahedral viruses (which range from about 20 nm to over 400 nm in diameter [21]) are orders of magnitude smaller than that of a human head, we can draw lessons from the indentation of the capsid structure for a helmet that may be impacted at any point on its surface. Secondarily, the capsid is constructed from multiple copies of relatively simple protein building blocks [22], and its wide range in sizes is accomplished by the larger viruses producing more copies of the protein building blocks and assembling them into larger structures with icosahedral symmetry. The form of the viral capsid structure inspired us to imagine a modular helmet in which multiple copies of relatively simple geometric building blocks are arranged icosahedrally to create helmets of different sizes, with the connections between the building blocks optimized to maximize the energy absorbed during impact.

The research described in this paper contributes to the field of work investigating the effects of curved structures under impact loading, which has implications for helmet designs and applications. Our long-term goal is to create complex three-dimensional (3D) structures using modular building blocks that incorporate an energy-absorbing interfacial material. In this paper, we study the effects of the suture morphology in relatively simple curved archway structures under quasi-static out-of-plane loading as a first step toward optimizing the suture design for helmet applications. Our study seeks suture geometries that minimize the degradation of the toughness and stiffness of the structure compared with a solid curved archway without sutures. Future studies will then focus on maximizing the toughness of the structure by incorporating an energy-absorbing interfacial material. We used finite element models (FEMs) to quantify the effects of the suture tab radius, contact angle, and the size of the tangent length when a dovetail-like feature was included in the suture morphology.

## 2. Materials and Methods

**Figure 1:**
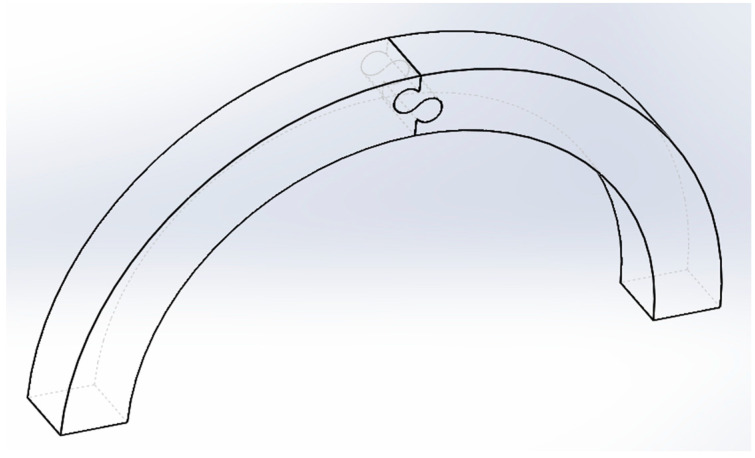
Representative archway pair with one full suture repeat.

While the intended helmet application is hemispherical, the specific size and shape of the individual pieces used to construct the helmet (e.g., modular building blocks such as those used in viral capsids or soccer balls [23]) adds additional variables that will be explored in future studies. In this study, pairs of simple archway structures with an inner radius approximately the size of an average head and thickness of a typical bicycle helmet liner [24] were created to isolate the effects of the suture geometry under out-of-plane loading. Archway pairs with an inner radius of 100 mm and a cross-sectional area of 25.4 mm × 25.4 mm were created with mirror-image suture geometries at the connecting face (Figure 1).

### 2.1. Suture Variation

**Figure 2:**
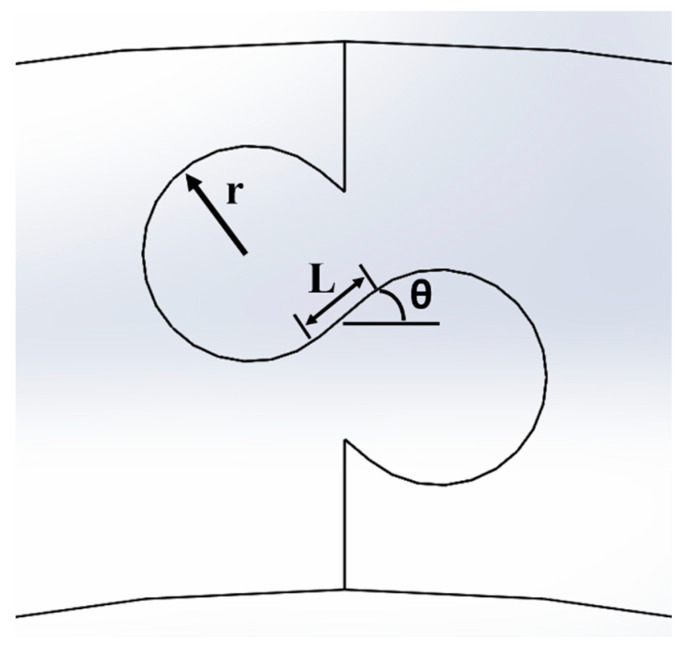
Three suture geometry parameters were varied: tab radius (r), contact angle (θ), and tangent length (L).

To create unique archway geometries, three suture geometry parameters were varied (Figure 2): the suture tab radius (r), ranging from 1 mm to 5 mm in 1 mm increments; the contact angle between the suture tabs (ϴ), ranging from 0° to 40° in 10° increments; and the length of the straight-line segment connecting the protruding and recessed tabs, termed the tangent length (L), ranging from 0 mm to 20 mm in 1–2.5 mm increments (the smaller increment was used in studies with large contact angles, as the maximum possible tangent length decreased with increasing contact angle). The structure has no tensile strength under pullout loading when a contact angle of 0° is used, as there is no geometric interlocking between the two archways; larger contact angles increase the tensile strength because of increased interlocking [17]. Introducing a tangent length creates dovetail-like suture tabs with a significantly larger contacting surface area, which has been shown correlate with increased strength and toughness [18].

**Figure 3:**
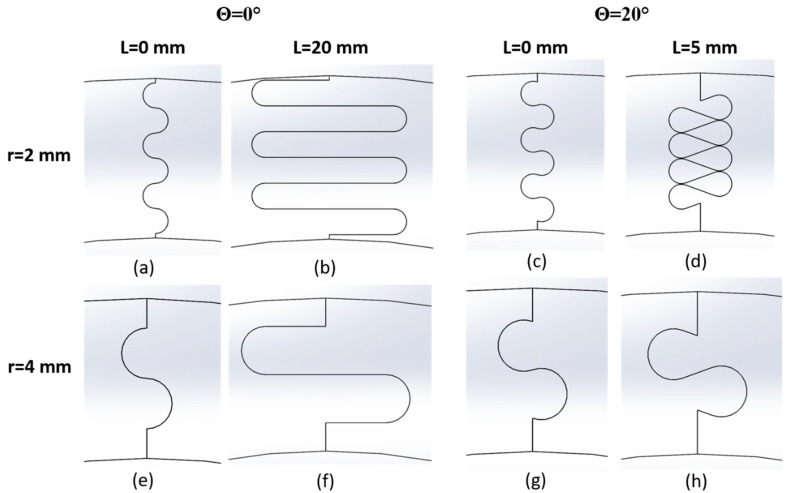
Suture shapes produced as the contact angle (**a**,**b**,**e**,**f** vs. **c**,**d**,**g**,**h**), tangent length (**a**,**c**,**e**,**g** vs. **b**,**d**,**f**,**h**), and tab radius (**a**–**d** vs. **e**–**h**) are changed.

For this study, the outline of one full suture repeat (i.e., one protruding tab and one recessed tab) was duplicated in integer increments as many times as possible within the 25.4 mm thickness, to ensure that the structures with a smaller suture tab radius would benefit from the largest possible contact area. Additionally, a minimum of one full suture repeat (e.g., Figure 2) was used to ensure consistency between the geometries, which limited the maximum suture tab radius used. The suture geometry was mirrored about the mid-point in the height, where we expect zero stress under pure bending. Examples of different suture geometries produced as the three geometric parameters are varied are shown in Figure 3.

Not all parameter combinations in these ranges could be simulated. For instance, the suture outline created when L = 5 mm for r = 2 mm and θ = 20° (Figure 3d) was nearly overlapping, and increasing the tangent length further was not possible for this tab radius/contact angle combination. Overlaps in the suture outline were induced in sutures with a non-zero contact angle as the tangent length was increased; the effects were noticeable at small contact angles when the suture tab radius was small, and only at larger contact angles when the suture tab radius was large. Similarly, when the suture tab radius was very small, no tangent length could be introduced when the contact angle θ ≥ 30° without creating overlaps in the outline. Cases that produced only one data point for a given specific tab radius/contact angle combination were not included in the study.

### 2.2. Total Suture Length

**Figure 4:**
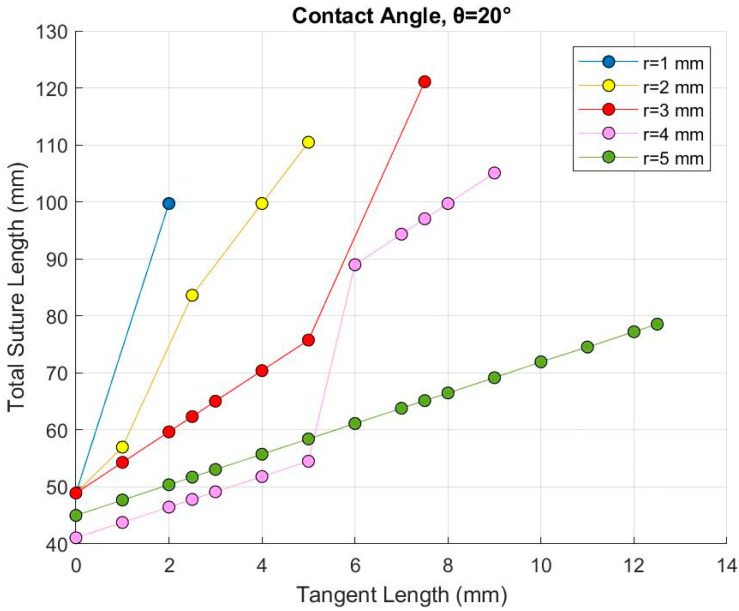
Representative example of the relationship between total suture length (global variable) and suture tab radius and tangent length, for a contact angle of 20° (the three local variables). While the three local variables are interchangeable (i.e., the same data can be graphed using different variables on the *x*-axis), plotting tangent length on the *x*-axis is the easiest to interpret, since L has the largest range of variation.

This study investigates how variations in the suture parameters affect the mechanical response of the archway. However, because the number of suture repeats that could be included within the thickness of the archway depended strongly on the local suture parameters, we examined the structural results as a function of the total suture length (length of the pathway from inner to outer diameter of the archway), which is a global variable. The total suture length for a given suture geometry encapsulates the underlying geometric properties of suture tab radius, tangent length, contact angle, and the number of suture repeats. Figure 4 shows a representative figure illustrating the relationship between the suture parameters and the resulting total suture length for all geometries with a contact angle of 20°; similar trends were observed in the other contact angles. Increasing the contact angle decreases the total suture length—i.e., a steeper contact angle occupies more vertical space per suture tab, allowing fewer repeats overall. 

For a single suture repeat, a larger radius leads to a longer total suture length when holding the contact angle and tangent length constant. However, Figure 4 shows that increasing the tab radius at a given tangent length (compared along a vertical line) generally decreases the total suture length due to the smaller number of repeats that can fit in the archway at large radii. This suggests that the number of repeats drives the contact area more than the tab radius alone. Similarly, for a single suture repeat, increasing the tangent length when holding the contact angle and tab radius constant leads to an incremental increase in the suture length by elongating the suture tab horizontally. However, increasing the tangent length also compresses the suture tabs vertically when the contact angle is greater than 0°, which allows for more suture tabs to fit in the archway, causing a significant increase in the total suture length when another suture tab repeat can be accommodated. For instance, Figure 4 shows that for a suture tab radius of 4 mm, increasing the tangent length increases the total suture length incrementally, until the tab has been compressed enough (at L = 6 mm) to add another suture repeat (from one to two repeats), which significantly increases the total suture length. Conversely, at a tab radius of 5 mm, increasing the tangent length increases the suture length incrementally, but there are no sudden jumps in the data, as there is no room for additional repeats to appear. This “added tab” phenomenon also explains why the total suture length is longer for r = 5 mm than for r = 4 mm when L ≤ 5 mm, as only one suture repeat was used in both cases, and the suture tab radius is the dominant property.

While the three local geometric parameters were the only dependent variables in this study, the effects of geometry on global mechanical behavior are described below in terms of total suture length. Using total suture length as the dependent global variable moving forward allows us to take the number of suture repeats into account—which was inconsistent between geometries—and provides a more direct relationship to the contact area than the suture’s geometric parameters at a local level would for investigating the mechanical response of the entire archway. Because the suture shape was extruded uniformly to create the archway piece, the total suture length can be used as a proxy for the total contact area.

### 2.3. Model Setup

**Figure 5:**
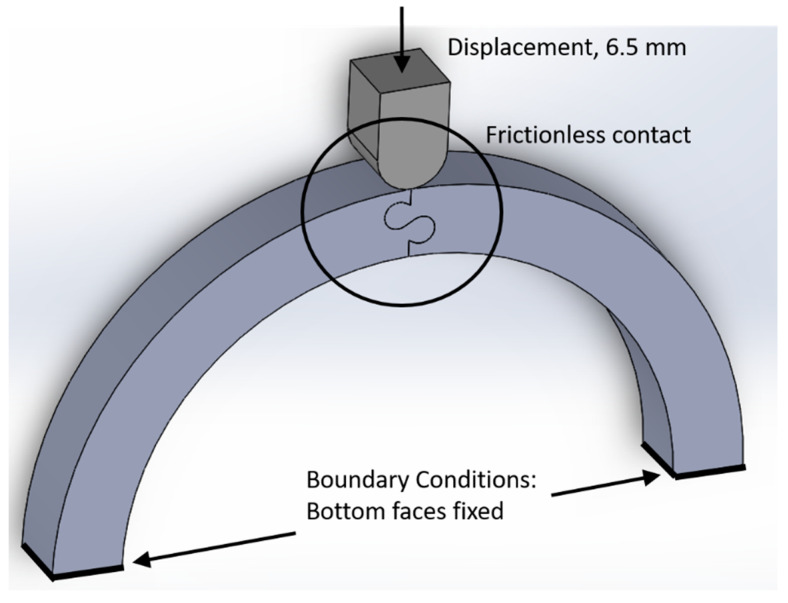
FEM setup: indenter displaces 6.5 mm, the bottom archway faces are fixed, and the three contacting surface pairs are modeled with frictionless contact.

FEMs were created and solved in ANSYS Mechanical (V19.2, PA, US). The archway pieces were simulated using polylactic acid (PLA) material, which will be used to 3D-print the archways for subsequent experimental testing. The archway structure was compressed vertically using a stainless-steel indenter, while the bottom face of each archway piece was fixed in place (see Figure 5). In the static, displacement-controlled simulations, the indenter was displaced 6.5 mm in 0.5 mm increments. This total displacement was chosen because the resulting deformation of the structure produced significant suture separation at the bottom section of the suture (under tension) and allowed the separation process to be observed. We treated both materials as linearly elastic, as this study was focused on the behavior of the structure in the absence of material yield and failure. Contacting surfaces (between the archway surfaces in the suture, and between the indenter and the two archway pieces) were modeled using frictionless contact. Anything more than minimal friction has been found to reduce the strength of the suture under tension because of the increased contact forces in the suture, which significantly contribute to tab fracture [17,18]. Because this study focuses on stiffness and toughness rather than strength, modeling the interfaces as frictionless provides a conservative estimate of mechanical behavior. A brief analysis regarding the addition of friction is described in the Discussion section.

A mesh convergence study was performed on a representative archway structure with suture parameter values in the middle of the intervals studied (θ = 20°, r = 3 mm, and L = 4 mm). A quadratic mesh with a global element size of 3 mm was used for all three components to create a coarse mesh. We added a contact sizing mesh control with an element size of 1 mm and 0.5 mm on all three contact surfaces to create a medium and fine mesh, respectively. Mesh parameters and output values of interest (see *Model Outputs* below for more details) are shown in Table 1. The model outputs increased less than 1% from the medium to fine mesh, indicating mesh convergence was achieved. We chose to use the fine mesh in all cases for more accurate results. For the representative archway structure, the number of nodes was 508,028 and the number of elements was 118,875; while slight changes to the number of nodes and elements occurred as the suture parameters were changed, this is an accurate representation of the average number of nodes and elements used in all studies. 

**Figure 6:**
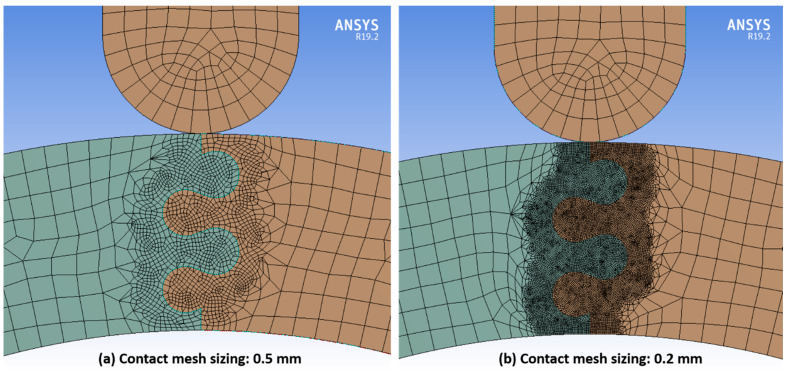
Mesh near the contacting surfaces of the suture for an archway with a contact angle of 20°, tab radius of 3 mm, and tangent length of 2 mm with (**a**) the initial fine contact mesh sizing of 0.5 mm, and (**b**) the updated extra-fine contact mesh sizing of 0.2 mm.

In several cases, the initial surface mesh was not fine enough to maintain contact separation between the suture surfaces, and significant overlap was observed between the suture tabs at large indenter displacements. In those cases, a contact sizing mesh control with a smaller element size of 0.2 mm was applied at all three contact surfaces. Figure 6 shows the initial and updated mesh for an archway with a suture contact angle of 20°, tab radius of 3 mm, and tangent length of 2 mm, which was one of the cases that required an extra-fine contact mesh. This modification produced meshes sufficiently fine enough to impede numerical issues with overlapping contact surfaces in all but one case (results from this case were not included in the study). Simulations that failed prior to displacing 6.5 mm were not included in the study.

### 2.4. Model Outputs

Two specific mechanical responses are of interest in this study: stiffness and toughness. In mechanics, the slope of a force versus displacement curve indicates the structure’s stiffness. Because all structures were displaced the same amount, in this study, we treat the normal contact force between the archway and the indenter at 6.5 mm of displacement (i.e., the final value) as analogous to the overall stiffness of the archway structure, which is a superposition of its contact stiffness and the bending stiffness. Subsequently, we refer to this value as the “final” contact force. While nearly all simulations produced linear contact force-displacement curves, in some cases, the contact force curve started to dip down at larger displacements; our data use the final contact force value at 6.5 mm, even if it was not the maximum in that case. No simulations produced non-linear contact force results that exceeded the linearly interpolated value at 6.5 mm. The toughness of the structure is captured by the total strain energy in the archway after the displacement is applied. As expected, the total strain energy increased with each time step. The outputs of final contact force (i.e., stiffness) and total strain energy (i.e., toughness) based on the parameterization of the suture characteristics suggest how the design of the suture geometry can alter the mechanical response normally governed by material properties. In this parametric study, we discuss the relative effects of suture geometries in detail; these outputs are not being used to predict absolute stiffness or toughness of each structure given the model simplifications described above (e.g., linear elasticity).

## 3. Results

**Figure 7:**
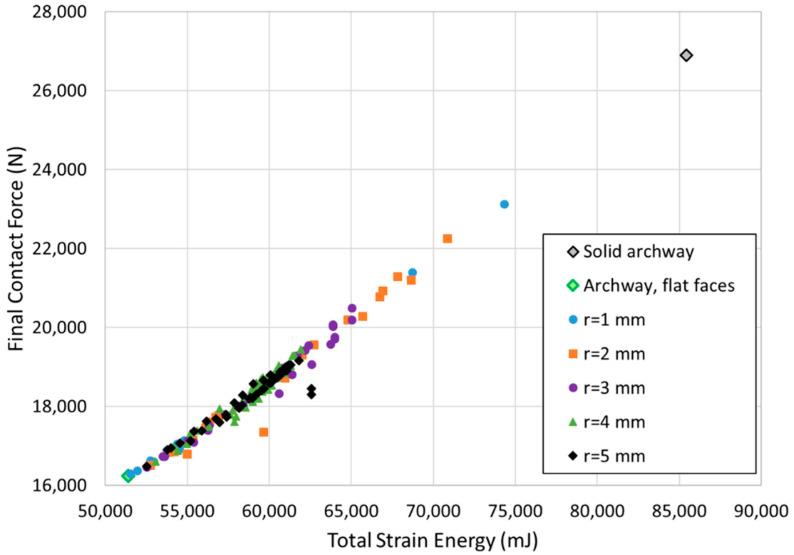
Final contact force vs. total strain energy for all cases.

Figure 7 shows all 163 successful simulations, grouped by suture tab radius, plotting final contact force against total strain energy. Our goal was to find suture geometries that allowed the structure to retain as much stiffness and toughness as possible, i.e., cases that fell high on both the *x*- and *y*-axis in Figure 7. The general trend showed that the final contact force increased roughly linearly with total strain energy. At smaller tab radii of 1–2 mm, the results spanned the entire range of output values; as the tab radius increased, the results were clustered more tightly in the lower ranges of output values. Our results show that the mechanical response degrades with increasing suture tab radius. 

**Figure 8:**
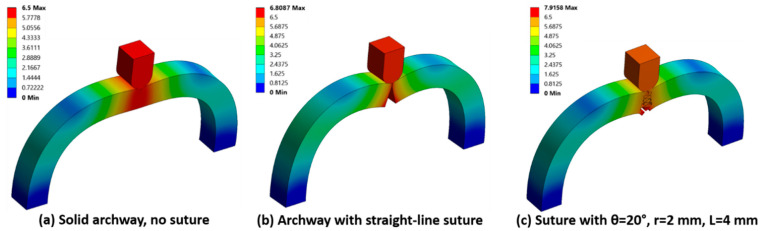
Total deformation (in mm) of the two base cases: (**a**) solid archway with no suture; (**b**) archway with a straight-line suture; and (**c**) one representative case with a dovetail suture. A scale factor of 3 was used to exaggerate the deformed shape. The maximum deformation in (**b**,**c**) exceeded the applied indenter displacement of 6.5 mm.

These results are compared to two base cases: (1) a solid archway with no suture, and (2) a two-piece archway with a straight-line suture, highlighted in Figure 7 and illustrated in Figure 8. The gray diamond in the top right corner of the graph indicates the results for the solid geometry with no suture; the archway with no induced flaws is stiffer and tougher than any of the archway pairs connected by sutures. This behavior is expected because the introduction of a suture allows the pieces to separate under tension, reducing the stiffness and toughness of the overall structure compared with the solid archway. The results in Figure 7 highlight suture parameter combinations that allow the structure to retain as much stiffness and toughness as possible. Introducing sutures into the structure allows an energy-absorbing material to be incorporated if a gap is introduced between the pieces, which will be included in future studies to further increase the toughness of the composite structure. The light green diamond near the bottom left corner in the graph indicates the results for the second base case, which has no interlocking strength under out-of-plane loading; as expected, this base case performed the worst of all geometric combinations. The best overall case is also identified in Figure 7.

The total archway deformation (i.e., resultant displacement) of the two base cases and one representative archway with a curved suture at the final indenter displacement of 6.5 mm is shown in Figure 8. Of the three, the smallest total deformation was observed in the solid archway, indicative of the stiffness of the structure. The largest total deformation in the cases with separate archway pieces was localized to the contacting archway surfaces and was highest at the bottom of the archway (which is the location experiencing the highest tensile load).

**Figure 9:**
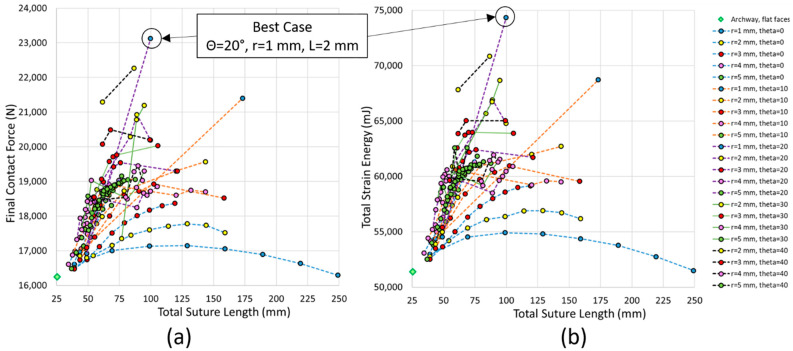
(**a**) Final contact force and (**b**) total strain energy plotted as a function of the total suture length in each archway pair.

To determine the effect of global geometric parameters on the resulting stiffness and toughness, the final contact force and total strain energy for all cases is plotted as a function of the total suture length in Figure 9. The trends in both stiffness and toughness were nearly identical: both tended to increase with increasing total suture length and contact angle. However, there were non-monotonic results in the dataset, and stiffer and tougher structures were produced by geometries with moderate total suture lengths and moderate contact angles. The geometric parameters that led to the optimal and least optimal responses are shown in Table 2. Perhaps surprisingly, the least optimal case had a tangent length of 17.5 mm instead of 20 mm (holding other variables constant). In comparison, the L = 20 mm case used one fewer tab repeat, producing a slightly stiffer and tougher structure with a total suture length of 236.8 mm; however, this geometry still ranked as the second least optimal case.

**Figure 10:**
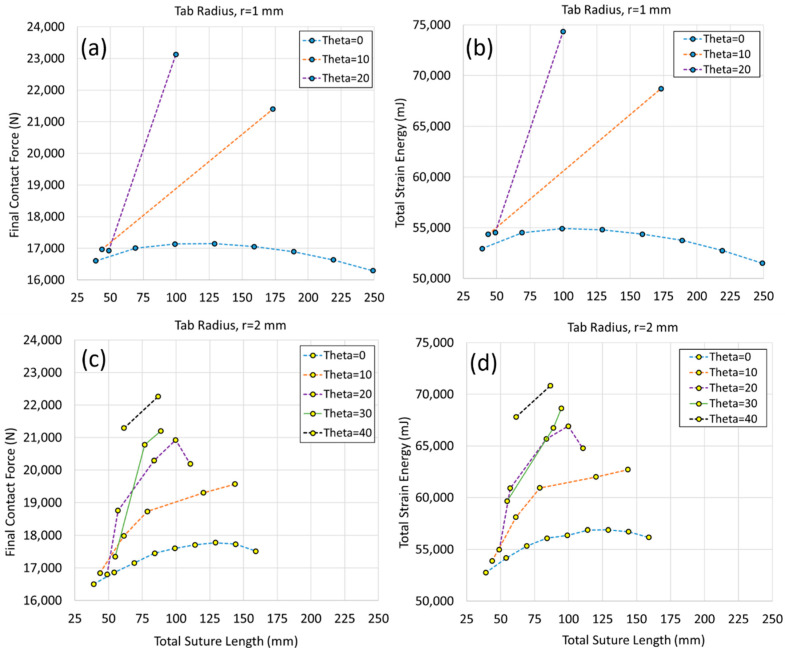
Final contact force and total strain energy for archways with a suture tab radius of 1 mm (**a**,**b**) and 2 mm (**c**,**d**) as a function of the total suture length.

Plotting subsets of the data elucidates the geometric trends. The final contact force and total strain energy as a function of total suture length are shown in Figure 10 for the archway structures with suture tab radii of 1 mm and 2 mm. The results for archway structures with suture tab radii of 3–5 mm (clustered together in the lower left portion of the plots in Figure 9) were mechanically suboptimal, and the results were not as sensitive to changes in the suture geometry as the archways with a smaller suture tab radius; therefore, these data are not plotted separately. 

The plots in Figure 10 (and in Figure 9, although harder to identify) indicate that, generally, both stiffness and toughness improved (increased) with the increasing contact angle and with increasing total suture length. That said, increasing the contact angle when the tangent length was 0 mm (i.e., the first data point in each series) did not appreciably stiffen or toughen the structure unless very large contact angles were used; only with contact angles of 30° or 40° (which were only possible for a suture tab radius ≥ 2 mm) did the increased suture tab interlocking significantly increase the stiffness and toughness of the archway structure. Similarly, increasing the tangent length when the contact angle was 0° for suture tab radii of both 1 mm and 2 mm did not appreciably stiffen or toughen the structure. In fact, after an initial slight increase in stiffness and toughness, they both started to drop as the total suture length was increased further. There is initially some structural benefit to increasing the contact area via the tangent length, since the protruding tabs near the bottom of the archway maintain contact longer than the tabs with no tangent length; however, that benefit is reduced as the overall stiffness of the protruding tabs decreases; they start to behave more like cantilever beams, which weaken substantially as their length is increased. A sharp decrease in stiffness and toughness was observed in the 2 mm tab radius case with a contact angle of 20°, as the tangent length increased from 4 mm to 5 mm (the total suture length increased from 99.7 mm to 110.5 mm, and the number of suture repeats did not change). Despite this seemingly slight change to the geometry, this significantly reduced the thickness of the material used to support the protruding side of the tab, resulting in a compromised structure. Therefore, there are limits to the usefulness of increasing the suture tangent length.

When the contact angle and total suture length were increased simultaneously, significant stiffness and toughness increases were observed. There are, of course, limits to the parametric combinations that can be used, as discussed previously. For example, in cases with suture tab radius of 1 mm, the allowable tangent lengths were quite small with any contact angle > 0° (though the small radius allowed for many tab repeats, resulting in moderate total suture lengths). Additionally, the contact angle could not exceed 20° without impinging upon physical limits. Despite the constraints on both the contact angle and the tangent length that could be used with these small suture tab radii, the stiffest and toughest archway structure was created with a tab radius of 1 mm, a contact angle of 20°, and a tangent length of 2 mm.

## 4. Discussion

The archway stiffness and toughness generally improved with the increasing contact angle (because of the increased interlocking between the suture tabs) and increasing tangent length (the increased interdigitation between the two pieces reduced the degree of separation in the bottom part of the archway, which was under tension). However, very large contact angles were necessary to provide substantial stiffness and toughness in cases where long tangent lengths could not be integrated into the suture geometry (due to physical limitations, e.g., the suture with a tab radius of 2 mm and contact angle of 40° could only physically allow tangent lengths of 0 mm and 1 mm). There were also limits to the effectiveness of maximizing the tangent length: in cases with a contact angle of 0°, slight drops in stiffness and toughness were observed with increased tangent length, as the suture tabs behaved more like cantilever beams, which weaken significantly with length. At non-zero contact angles, it was possible to create suture geometries with exceedingly thin (and therefore very weak) connective regions for the protruding tabs. 

Stiffness and toughness were optimal when the smallest suture tab radius was used (1 mm), in conjunction with the largest possible values of contact angle and tangent length. As described earlier, a small tab radius allowed for more suture repeats through the thickness of the archway, which appears to provide a mechanical benefit despite the total suture length not being at the upper end of the values produced in this study. For any given tab radius value with a contact angle of 0° (i.e., only varying tangent length), the number of suture repeats in the archway remained constant and no gains in stiffness or toughness were observed. This suggests that the improved response when the contact angle was >0° could not be attributed solely to the increased total suture length. In their analytical study, Malik and Barthelat (2018) studied the mechanical response of double- and multi-locking sutures under tension and found that the progressive pullout of multiple tabs produced a “geometric hardening” that increased the stiffness and toughness of the structure compared with the single suture tab [18]. While the out-of-plane loading in our study produced a more complex stress state, the lower half of the contacting suture surface was in tension, and the larger numbers of suture tab repeats meant a similar progressive pullout was required as the indenter displacement increased, resulting in a stiffer and tougher structure.

Finally, we compared the results from a representative case modeled with frictionless contact and with frictional contact with a coefficient of 0.2 to confirm that the addition of friction would reduce the strength of the structure under out-of-plane loading as it did under tensile loading [17,18]. We chose a suture geometry with parameter values in the middle of the intervals studied (θ = 20°, r = 3 mm, and L = 4 mm). The stiffness and toughness, as captured by the final contact force and total strain energy, respectively, increased by 2.73% and 0.53%, and the maximum von Mises stress increased by 11.1%. This indicates that the slight increases in stiffness and strength would be outweighed by the substantial degradation of the strength of the structure.

This study is only the first of many that are necessary to design a modular helmet optimized for high-speed impacts. Future studies will investigate modifications to the overall geometry (i.e., more complex structures than simple two-piece archways), while incorporating the optimal suture geometries found in this work. Initially, we will explore the effects of out-of-plane loading on the faces of the building blocks (as opposed to an edge, as in this study) by simulating archway pieces revolved 60° (i.e., connecting three pieces). Later, we will explore hemispherical structures with increased complexity using modular building blocks to study the effects of the morphologies of the individual shapes and all potential out-of-plane loading effects (e.g., on a vertex, which can only be tested in 3D). Further extensions in the model will be made at the material level, such as adding an interfacial soft tissue at the suture to optimize energy absorption. In the biological systems that inspired this work, the soft interfacial materials found in the small gaps in the suture region allow for substantial deformation and energy absorption. To complete the modeling phase of this work, high-speed impacts need to be simulated to confirm the optimal geometry and material combinations under conditions relevant for the final helmet application. Impact loading will produce stress waves that reflect off interfaces, and these dynamic effects must also be thoroughly quantified. The optimal geometry and material combinations found from the simulation results will be used to guide the construction and assembly of experimental test pieces for high-speed drop tests to predict the injury reduction provided by a particular helmet design.

## 5. Conclusions

Several structures found in Nature utilize a thin suture in between building blocks of a stiff material, which provide flexibility while upholding protection for biological organisms. We seek to better understand the geometries of these thin sutures and the mechanical benefits they can provide to the overall stiffness and toughness of the structure. In this study, we developed FEMs to explore the effect of suture geometry on the stiffness and toughness of an archway structure displacement loaded out-of-plane up to 6.5 mm. The final normal contact force allowed us to examine the stiffness of the archway, and the total strain energy allowed us to examine the toughness of the archway. Our results indicate that the stiffness and toughness were complex functions of the three local suture geometric variables: suture tab radius, contact angle, and tangent length. In particular, sutures with large contact angles and large tangent lengths generally led to stiffer and tougher structures, as these properties increased the amount of interlocking and interdigitation between the pieces, respectively. Sutures with a small tab radius exhibited the most sensitivity to the input parameters, and the stiffest and toughest archways were found when the smallest suture tab radius of 1 mm was used. Results suggested that it was a combination of the number of tab repeats with the largest possible contact surface area that improved the mechanical response of the archway structure. The study revealed several suture geometries that hold significant promise, which can aid in the development of hemispherical 3D structures for dynamic impact applications, such as helmet designs.

## Figures and Tables

**Table 1:** Mesh convergence results obtained from a representative archway structure (θ = 20°, r = 3 mm, and L = 4 mm). Quadratic elements were used in all three cases. The percent change indicates the percent change in the model output compared with the previous mesh (i.e., from coarse to medium and from medium to fine).

	Mesh Parameters	Nodes (Qty)	Elements (Qty)	Final Contact Force (N)	% Change	Total Strain Energy (mJ)	% Change
Coarse	Global element size 3 mm	53,114	11,133	18,878.2	N/A	59,355	N/A
Medium	Global element size 3 mm, contact sizing 1 mm	177,144	39,784	19,388.0	2.70%	61,695	3.94%
Fine	Global element size 3 mm, contact sizing 0.5 mm	508,028	118,875	19,423.4	0.18%	62,171	0.77%

**Table 2:** Geometric parameters of best- and worst-performing cases.

Parameter	Optimal Case	Least Optimal Case
Contact angle	20°	0°
Tab radius	1 mm	1 mm
Tangent length	2 mm	17.5 mm
Number of suture repeats	8 repeats	6 repeats
Total suture length	99.7 mm	249.1 mm
Final contact force	23,124 N	16,295 N
Total strain energy	74,331 mJ	51,494 mJ

## Data Availability

The data presented in this study are openly available in FigShare [25].
